# Descriptive epidemiology of measles in the southern region of Afghanistan, 2024-2025: a secondary analysis from regional case-based surveillance data

**DOI:** 10.1016/j.ijregi.2026.101036

**Published:** 2026-10-04

**Authors:** Shoaib Naeemi, Mustafa Rahimi, Nisar Ahmad Niazi, Rohullah Sakhi

**Affiliations:** 1 Eastern Mediterranean Public Health Network, Afghanistan Country Office, Kabul, Afghanistan; 2 Faculty of Public Health, Kabul University of Medical Sciences, Kabul, Afghanistan; 3 TPM Technical Department, Particip GmbH Afghanistan Country Office, Kabul, Afghanistan; 4 Faculty of Medicine, Afghan International Islamic University (AIIU), Kabul, Afghanistan

**Keywords:** Measles, Descriptive epidemiology, Case-based surveillance, Afghanistan, Measles vaccine, Eastern Mediterranean region

## Abstract

**Objectives:**

After a three-fold increase in measles outbreaks in Afghanistan during 2024, this study aimed to assess the descriptive epidemiology of measles in the southern region.

**Methods:**

This descriptive cross-sectional study was conducted on case-based measles surveillance data retrieved from the District Health Information System 2 for the period from January 1, 2024 to December 31, 2025. Demographic, clinical, and immunization status data were summarized descriptively and surveillance system performance was assessed using World Health Organization indicators.

**Results:**

Of 4379 suspected measles cases, 3346 (76.4%) were laboratory-confirmed and 52.7% were males. The median age was 24 months (interquartile range 12-60), and four-fifths were children aged under 5 years. A total of 66.5% of cases were from Kandahar; overall, 80.9% of all cases and 80.2% of confirmed cases did not have a measles-containing vaccine record. Helmand had the highest proportion of unvaccinated cases at 97.7%. The overall discarded rate was 10.55 per 100,000 population; the adequate time for investigation, specimen adequacy, laboratory receipt of specimen, and communication of results with public health authorities were 98.3%, 99.1%, 77.7%, and 82.8%, respectively.

**Conclusion:**

Measles transmission in southern Afghanistan during 2024-2025 was predominantly recorded along with a large unvaccinated population for measles-containing vaccine, concentrated in children aged under 5 years, with highlighted provincial disparities in immunization and surveillance timeliness. Geographically differentiated responses prioritizing outreach vaccination and strengthening specimen transport in the lowest-performing provinces are urgently required.

## Highlights

• A total of 4379 measles cases analyzed across six provinces of southern Afghanistan, 2024-2025.
• A total of 76.4% of suspected measles cases were laboratory-confirmed.
• Nearly four-fifths of confirmed cases occurred in children aged under 5 years.
• Cases with no reported vaccination history were documented in over 80% of confirmed measles cases.
• The surveillance performance met the World Health Organization–recommended targets in discarded rate, adequacy of investigation, specimen adequacy, and communication of results with public health authorities, whereas receipt of specimen to laboratory within the recommended time fell short of the target.

## Introduction

Measles, which is caused by a single-stranded ribonucleic acid virus of the family Paramyxoviridae, is known for centuries to mankind, characterized by its rapid spread (reproduction number estimated between 12 and 18), high fever, coryza, cough, conjunctivitis, maculopapular rash with the potential of progressing to severe complications such as pneumonia, blindness, and brain damage [1]. Before measles vaccine introduction during the 1960s, an approximate 90% of children contracted this infection before reaching 10 years of age, making its prevalence near-universal [2,3]. So far, substantial progress has been achieved in reducing the global burden of this infection with an estimated decline of over 70% in number of cases between 2000 and 2024 [4].

Despite global efforts, measles claim nearly 100,000 deaths annually [1]. With emergence of coronavirus disease 2019 (COVID-19) and country-level challenges, measles peaked once again during 2024, marking the highest reported since 2003 [2,[4], [5], [6]]. On the other hand, measles has a disproportionate effect on low- and middle-income countries, particularly among those experiencing conflicts, displacement, and fragile health systems, with a mean case-fatality rate reaching over 2%, and has the highest incidence among children aged under 2 years [7]. Susceptibility of these settings is exacerbated by burden of malnutrition, which compromises immunity among children, which serves as one of the preliminary steeping stones for measles resurgence [8]. Among other regions worldwide, the Eastern Mediterranean Region (EMR) is experiencing an acute phase, with nearly 150,000 cases reported from this region between 2019 and 2024. In this region, 70% of cases were either unvaccinated or did not receive the full dose. The onset of COVID-19 pandemic expanded the pool of zero-dose children in EMR with Afghanistan and Pakistan accounting for more than 6 million of this pool, practically making these countries the epicenter of EMR for unvaccinated children [9].

Afghanistan exemplifies the compounding influence of conflict and immunization with fluctuating trends elucidating its progress in reaching universal routine immunization coverage. During 1999-2006, Afghanistan experienced a substantial growth in routine immunization but dramatically faced a declining pattern after 2019 [6]. The COVID-19 pandemic exerted further pressure on an already struggling system, with routine immunization being as one if its immediate consequences [10]. On the other hand, rapid political transitions in August 2021 jeopardized the financing of health system, with inevitable and cascading impacts [11]. Among other surging challenges, measles poses a significant challenge, raising to 200% of infected cases during 2022-2023 compared with 2019, with an almost-universal burden among children aged under 5 years [12]. The inclining pattern continued in 2024, with more than 400 outbreaks reported in 2024 compared with 127 of 2023. In response to this challenge, the National Expanded Program on Immunization with the United Nations Children’s Fund, the World Health Organization (WHO), and Gavi launched a nationwide campaign of measles-containing vaccine (MCV) in two phases, reaching to over 16 million children across all provinces, focusing on cold-climate and hot-climate provinces [13]. The Multiple Indicator Cluster Survey (MICS) 2022-2023 showed 51% of measles containing vaccine does 1 (MCV1) coverage [14], whereas the routine MCV1 provision alone was found to be insufficient to reach population immunity threshold, elucidating the need for further supplementary immunization activities (SIAs) [15].

Although the national burden of measles is significant, sub-national and region-wise epidemiologic characterization of the cases remains barely investigated. In this regard, the southern region of Afghanistan, consisting of six provinces, Kandahar, Helmand, Urozgan, Farah, Nimroz, and Zabul, reported the highest number of cases of measles during 2019-2023 [12]. Moreover, the MCV1 coverage for provinces in this region was reportedly lower than 50% (except for Nimroz with 59%) in MICS 2022-2023 [14], whereas the modeled estimates of coverage-adjusted transmission potential (Rcov) reported that all six provinces in the southern region exceeded the epidemic transmission threshold, putting them in highest-risk provinces with potential of measles virus transmission [15]. The region is aligned with the national scheme of health services delivery of Basic Package of Health Services (BPHS) and Essentials Package of Hospital Services (EPHS), with routine immunization services provision integrated from the basic levels of this system. MCV is administered in two doses for children at 9 and 18 months of age as part of national routine schedule [16]. Considering the established burden of the measles in this region, generating evidence on epidemiologic profile of measles cases in this region along with assessing the measles case-based surveillance performance and vaccination status of cases would help in articulating tailored and epidemiologically sound interventions to reduce the burden of the cases in this region. In this study, the authors aimed to describe the epidemiologic profile, vaccination status, and surveillance system performance of measles cases in a 2-year frame.

## Methods

### Study design

This was a descriptive cross-sectional study conducted through a secondary analysis of case-based measles surveillance data retrieved from the District Health Information System 2 database of the National Diseases Surveillance and Response Department of the Ministry of Public Health of Afghanistan (MoPH) for the period from January 1, 2024 to December 31, 2025. December 31, 2025 marks the database extraction cut-off date rather than the final case-onset date.

### Study setting

Six provinces are located administratively in the southern region (Kandahar, Helmand, Urozgan, Zabul, Farah, and Nimroz), spanning across 235,000 km^2^ of arid and semi-arid terrains and neighbors with Pakistan (east) and Iran (west). The total population of this region based on recent National Statistics and Information Authority (NSIA) estimate reaches 4,667,280, with province-wise populations as the following: Kandahar 1,471,666, Helmand 1,525,188, Urozgan 477,593, Zabul 405,050, Farah 593,841, and Nimroz 193,942 [17]. Kandahar is the center for the region’s health services and hosts Kandahar Regional Reference Laboratory (KRRL) as the diagnostic center of National Surveillance and Response (NDSR) in the region. Health services in this region is aligned with the national platform of BPHS and EPHS, with primary care provided by health posts, mobile health teams, basic health centers, comprehensive health centers, and district hospitals while provincial hospitals and Mirwais Regional Hospital act as referral hubs. Overall, this region has 371 health facilities scattered across all of the mentioned tiers of BPHS, with 72 serving as sentinel sites for surveillance. The KRRL provides laboratory support for measles and rubella serology for the southern region.

### Measles case-based surveillance system in southern Afghanistan

The measles case-based surveillance operates under the NDSR department of the MoPH. This surveillance system operates among the selected sentinel sites within each province to carry out measles surveillance and does not cover all the health facilities of each province. A patient with fever and rash presents to the outpatient department of a health facility; after a clinical assessment by the attending medical doctor, surveillance teams cross-reference the signs and symptoms of the patient with the case definition of measles. A suspected measles case is defined as “any person with fever and maculopapular rash across entire body and having at least one of the following symptoms: cough, coryza, conjunctivitis, or any individual clinically identified by medical doctor as suspected measles case.”

If the case meets the clinical case definition, it is immediately entered into the measles line list and classified as an alert case. Variables on the line list will be completed for each case and immediate contact tracing is initiated to seek out the potential outbreak.

A venous blood specimen is collected at the health facility at the time of clinical assessment and transported to the KRRL for serological testing along with a specific laboratory form. Specimens are tested using enzyme-linked immunosorbent assay for measles immunoglobulin M (IgM) antibodies on all specimens. Rubella IgM testing is additionally performed in cases in whom measles IgM is negative or equivocal or where rubella is clinically suspected. Once the laboratory result is received, the line list is updated with the following scenarios:

1. Laboratory-confirmed measles: IgM-positive for measles antibody for a suspected case who did not receive measles vaccine in the last 28 days,
2. Discarded: IgM-negative with adequate specimen for measles,
3. Laboratory-confirmed rubella: IgM-positive for rubella antibody, and
4. Equivocal: Measles IgM yielded was in the borderline and should be correlated with clinical picture and, if possible, retake the specimen.

The line list is updated with the laboratory classification. Case follow-up is subsequently conducted by the surveillance team to ascertain the final outcome of each reported case, which is categorized as (i) recovery, (ii) death, or (iii) referral with complications to a higher-level health facility (Supplementary Figure 1).

### Data source and variables

Data were extracted from the NDSR District Health Information System 2 at the MoPH. The data set included all suspected measles cases reported from the six southern provinces’ surveillance sentinel sites during the study period. The following variables were extracted: (i) demographic characteristics: age in months, sex, province of residence, and district of residence; (ii) clinical presentation: presence of fever, maculopapular rash, cough, coryza, and conjunctivitis; (iii) vaccination history: number of previous MCV doses received; (information regarding other vaccines received as part of routine immunization for the suspected case of measles is not recorded because the case investigation form only requires MCV vaccination history. Moreover, verification of vaccination is performed by card or recall, with varying practices within each health facility. For case investigation forms where the input in MCV history was missing or left blank, the vaccination status was addressed as unknown); (iv) surveillance and laboratory variables: date of rash onset, specimen condition, date of specimen receipt at the laboratory, measles IgM result, rubella IgM result, and final case classification; and (v) case outcome: recovery, death, or referral with complications. The extracted data were then reviewed for data completeness, implausible records (e.g. extended timeliness values), and other issues; a clean version of data was then used for further analysis.

### Data analysis

Descriptive statistical analysis was performed after data cleaning and management. Categorical variables, including sex, age group, province, vaccination status, clinical signs and symptoms, laboratory results, final classification, and case outcomes, were summarized as absolute frequencies and proportions. The continuous variable, age, was summarized using median and interquartile range (IQR) given its non-normal distribution. The performance of the surveillance system for measles and rubella was assessed based on the WHO-recommended indicators [18] (Table 1).

**Table 1:** WHO-recommended indicators for assessing measles and rubella surveillance system.

Indicator	Target
1. Percentage of surveillance units reporting to the next highest level on time, even in the absence of the case	≥80%
2. Annual reporting rate of discarded measles and rubella cases	≥2/100,000 population
3. Percentage of suspected measles and rubella cases that have had (i) an adequate investigation and (ii) initiated ≥80% within 48 hours of notification	≥80%
4. Percentage of suspected cases with adequate specimens for detecting acute measles and rubella infection collected and tested, excluding epidemiologically linked cases from the denominator	≥80%
5. Percentage of laboratory-confirmed outbreaks with samples adequate for detecting measles virus collected and tested in an accredited laboratory.	≥80%
6. Percentage of specimens received at the laboratory within 5 days of collection	≥80%
7. Percentage of IgM results reported to public health authorities by the laboratory within 4 days of specimen receipt	≥80%
8. Percentage of confirmed cases for which source of transmission is classified as endemic, imported, or importation related	≥80%

Abbreviations: IgM, immunoglobulin M; WHO, World Health Organization.

In indicator 3, adequate investigation referred to the collection of all of the following data elements from each suspected measles or rubella case: name or identifiers, place of residence, place of infection (at least to the district level), age (or date of birth), sex, date of rash onset, date of specimen collection, measles-rubella vaccination status, dates of all measles-rubella or measles-mumps-rubella vaccinations, date of notification, date of investigation, and travel history.

The data set was then checked for the feasibility of assessing the mentioned indicators, focusing on how much the data allows the calculation of the indicators. Consequently, indicators 1, 5, and 8 were removed from the final analysis.

A total of 13 records with biologically implausible timeliness values (<0 or >30 days between rash onset and laboratory receipt) were identified and excluded from timeliness analyses (n valid = 4366). All analyses were performed using Stata statistical software version 18 (StataCorp, College Station, TX, USA), whereas MS Excel 2016 (Microsoft Corporation, Redmond, WA, USA) was used to prepare the relevant graphs.

### Ethical considerations

This study involved a secondary analysis of routinely collected, de-identified national surveillance data. No primary data collection was conducted and no direct patient contact occurred. Individual patient consent was not required given the passive, secondary nature of the analysis and the de-identified format of the data set. Data access and use were authorized by the NDSR department of the MoPH. The study was conducted in adherence to the principles of the Declaration of Helsinki.

## Results

### Sociodemographic characteristics

Between January 1, 2024 and December 31, 2025, a total of 4379 suspected cases of measles were reported through case-based surveillance from six provinces in the southern region of Afghanistan. Suspected cases were nearly evenly split across both years: 2025 accounted for 2350 cases (53.7% of 2 years total) compared with 2029 cases in 2024. Overall, 2309 (52.7%) cases were male compared with 2070 (47.3%) female cases, with slightly higher number of reported cases among males in 2025 than in 2024 (Table 2). The median age of suspected cases was 24 months (IQR = 12-60 months), with almost four-fifths of suspected cases observed in age category of under 5 years. Children aged under 2 years were highly affected during study period, contributing to 41% of the overall cases. Moreover, nearly one in four suspected cases came from age categories of 60 months and beyond. Based on geographical distribution, two-thirds of cases were reported from Kandahar province during 2024-2025, whereas Nimroz had the lowest number of cases, with 33 (0.8%) (Figure 1). Number of suspected cases in Kandahar increased from 1235 (60.9%) in 2024 to 1675 (71.3%) in 2025, whereas Urozgan experienced a nearly two-fold decrease in 2025, with 216 (9.2%) suspected cases compared with 345 (17%) in 2024. A slight decrease in the number of suspected cases was also observed in Helmand province from 297 (14.6%) in 2024 to 257 (10.9%) in 2025. The reported suspected cases in remaining three provinces stayed almost similar across both years.

**Table 2:** Sociodemographic characteristics of suspected measles cases by year, southern region of Afghanistan, 2024-2025 (N = 4379).

Characteristic	2024 (n = 2029)		2025 (n = 2350)		Total (n = 4379)	
	n	%	n	%	n	%
Sex						
Male	1059	52.2	1250	53.2	2309	52.7
Female	970	47.8	1100	46.8	2070	47.3
Age group						
0-9 months	279	13.8	348	14.8	627	14.3
10-23 months	504	24.8	667	28.4	1171	26.7
24-60 months	807	39.8	878	37.4	1685	38.5
>60 months	439	21.6	457	19.4	896	20.5
Median age, months (IQR)	30 (12-60)		24 (12-60)		24 (12-60)	
Province						
Kandahar	1,235	60.9	1,675	71.3	2,910	66.5
Urozgan	345	17	216	9.2	561	12.8
Helmand	297	14.6	257	10.9	554	12.7
Zabul	133	6.6	140	6	273	6.2
Farah	11	0.5	37	1.6	48	1.1
Nimroz	8	0.4	25	1.1	33	0.8

Abbreviation: IQR, interquartile range.

**Figure 1:**
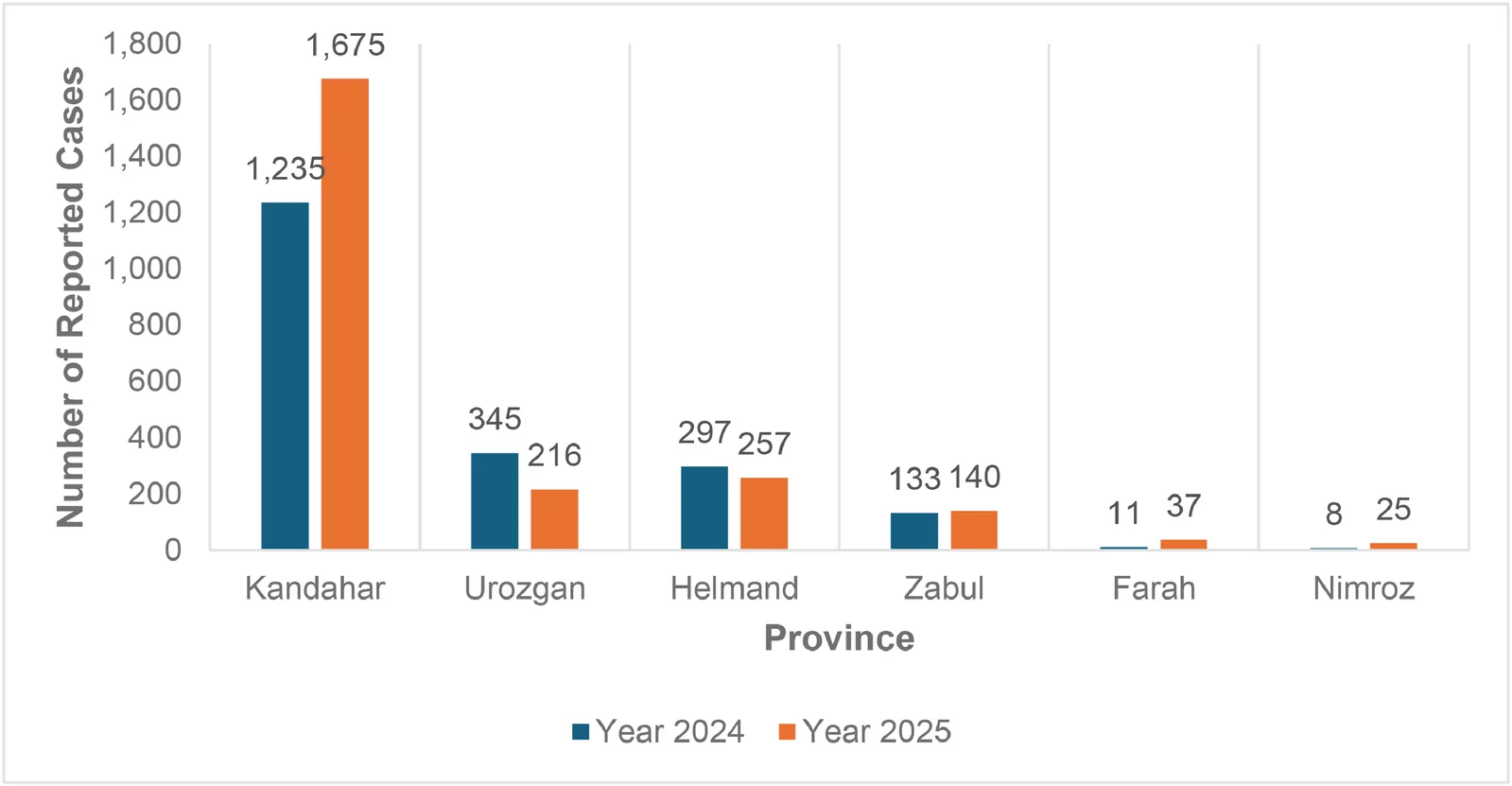
Provincial distribution of suspected measles cases by year, southern region of Afghanistan, 2024-2025.

### Clinical characterization and temporal analysis of cases

From the 4379 suspected cases during 2024-2025, 3346 (76.4%) were laboratory-confirmed measles, followed by 23 (0.5%) cases of confirmed rubella (Table 3). Overall, cough, coryza, fever, maculopapular rash, and conjunctivitis were the leading signs and symptoms. In total, the specimen adequacy proportion was 99.9%, whereas the presence of rubella was also tested. In testing for measles IgM, 19 (0.4%) were equivocal, whereas this proportion was only 0.2% (2 cases) in the rubella IgM results. Moreover, 985 (22.5%) of the suspected cases were discarded.

**Table 3:** Clinical and laboratory characteristics of suspected measles cases, southern region of Afghanistan, 2024-2025 (N = 4379).

Characteristic	n	%
Signs and symptoms		
Fever	4376	99.9
Maculopapular rash	4377	99.9
Cough	4379	100
Coryza	4379	100
Conjunctivitis	4369	99.8
Specimen condition		
Adequate	4374	99.9
Inadequate or specimen not collected	5	0.1
Measles IgM result		
Positive	3346	76.4
Negative	1009	23
Equivocal	19	0.4
Specimen not collected	4	0.1
Rubella IgM result^a^ (n = 994 tested)		
Positive	23	2.3
Negative	969	97.5
Equivocal	2	0.2
Final classification		
Laboratory-confirmed measles	3346	76.4
Discarded (IgM-negative)^b^	985	22.5
Laboratory-confirmed rubella	22	0.5
Final outcome^c^ (n = 925 with recorded outcome)		
Recovered	920	99.5
With complications	5	0.5
Outcome not recorded (missing)	3454	78.9

Abbreviation: IgM, immunoglobulin M.

a Rubella IgM tested in 994 specimens (22.7% of total); the remaining 3385 specimens were not tested for rubella. Percentages for rubella IgM rows use n = 994 as the denominator.

b Discarded cases had an adequate specimen that tested IgM-negative for both measles.

c Outcome was recorded for 925 cases (21.1%); percentages in this section use n = 925 as the denominator.

The temporal pattern of suspected measles cases differed markedly between the 2 years (Figure 2): 2024 showed a gradual, sustained rise from January through a July peak (12.7% of that year’s cases), whereas 2025 cases were concentrated in a January-to-April plateau, peaking in March (14.5% of that year’s cases), followed by a sharp decline from May and a pronounced drop in June, with a modest secondary rise in November (full monthly figures are shown in Supplementary Table 1). On an annual basis, the highest number of suspected cases, 258 (12.7%), was reported during July in 2024, whereas the southern region experienced the highest number cases during March 2025 with 341 (14.5% of the 2025 overall cases), making it the hardest-hit month in 24 months of the study period. A seasonal contrast was observed in both years, with a higher number of reported cases in summer during 2024, whereas late winter and early spring had the highest number of cases in 2025. However, on an overall basis, almost 30% of cases came from the winter season (January-March), followed by spring (April-June) with a proportion of 27%, regardless of comparative differences.

**Figure 2:**
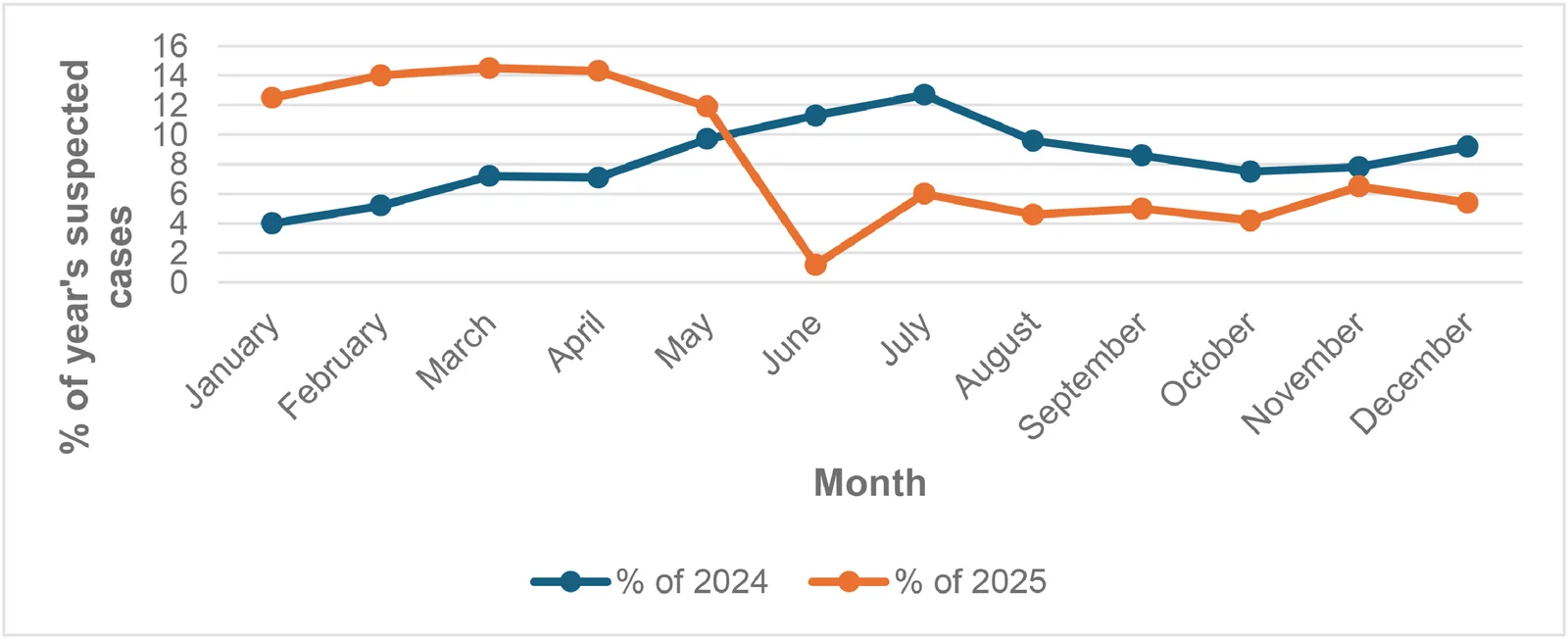
Suspected measles trend during 2024-2025 (N = 4379).

### Vaccination history of measles suspected cases in the southern region

Among 4379 suspected measles cases in 2024-2025, four-fifths, (3541, 80.9%) did not receive the MCV, whereas only 116 (2.6%) received two or more MCV doses (Table 4). Moreover, 380 (8.7%) suspected cases were in the undue vaccination category. Interestingly, 2683 (80.2%) confirmed measles cases did not receive the MCV vaccine and only 94 (2.8%) of them received two or more doses of the measles vaccine. Based on geographical distribution, the highest proportion of children with no MCV vaccine history was reported from Helmand (97.7%), followed by Urozgan (94.5%) and Zabul (94.1%). In contrast, the proportion of individuals receiving at least one dose of the measles vaccine was high in Kandahar, with 319 (11%). Based on age categories, only 296 (16.5%) suspected cases received at least one dose of the measles vaccine, whereas 58.6% of children aged under 2 years showed no history of receiving an MCV dose. Moreover, nearly all individuals (96.4%) aged older than 2 years did not receive the vaccine at all. The vaccination profiles of males and females remain almost similar; 80.3% and 81.5% did not receive the MCV, respectively. In addition, only 7.3% of males and 7.8% of females received at least one dose of the measles vaccine.

**Table 4:** MCV vaccination status of suspected measles cases by province, final classification, age group, and sex, southern region of Afghanistan, 2024-2025.

Subgroup	Total	No MCV history	Undue vaccination^a^	1 dose	≥2 doses	Unknown^b^
	n	n (%)	n (%)	n (%)	n (%)	n (%)
Total (all cases)	4379	3541 (80.9)	380 (8.7)	331 (7.6)	116 (2.6)	11 (0.3)
Province						
Kandahar	2910	2142 (73.6)	326 (11.2)	319 (11.0)	112 (3.8)	11 (0.4)
Urozgan	561	530 (94.5)	25 (4.5)	6 (1.1)	0 (0.0)	0 (0.0)
Helmand	554	541 (97.7)	11 (2.0)	1 (0.2)	1 (0.2)	0 (0.0)
Zabul	273	257 (94.1)	9 (3.3)	5 (1.8)	2 (0.7)	0 (0.0)
Farah	48	41 (85.4)	6 (12.5)	0 (0.0)	1 (2.1)	0 (0.0)
Nimroz	33	30 (90.9)	3 (9.1)	0 (0.0)	0 (0.0)	0 (0.0)
Final classification						
Confirmed measles	3346	2683 (80.2)	301 (9.0)	259 (7.7)	94 (2.8)	9 (0.3)
Discarded	985	819 (83.1)	77 (7.8)	66 (6.7)	21 (2.1)	2 (0.2)
Age group						
Under 2 years	1798	1053 (58.6)	379 (21.1)	296 (16.5)	69 (3.8)	1 (0.1)
Over 2 years	2579	2487 (96.4)	1 (0.1)	34 (1.3)	47 (1.8)	10 (0.4)
Sex						
Male	2309	1854 (80.3)	221 (9.6)	169 (7.3)	61 (2.6)	4 (0.2)
Female	2070	1687 (81.5)	159 (7.7)	162 (7.8)	55 (2.7)	7 (0.3)

Abbreviation: MCV, measles-containing vaccine.

a “Undue vaccination” denotes infants who received MCV before the recommended minimum age of 9 months; vaccination at this age is not considered immunologically protective. These infants are classified separately from “zero dose” because they did receive a dose but outside the eligible age window. Virtually all undue vaccination cases (99.7%) occurred in the 0-22 month age group (median age: 6.8 months). The absence of undue vaccination in age groups ≥23 months is expected.

b In case investigation forms of the measles suspected cases, the MCV vaccination status has not been mentioned or left blank.

### Measles surveillance system performance

The performance of the measles surveillance system was assessed separately for both years against the WHO indicators. The annual discarded rate was 11.29 and 9.81 for 2024 and 2025, respectively, about five times higher than the target of two per 100,000 population (Table 5). Of six provinces, only Farah and Nimroz performed lower in this indicator, with the highest rate reported from Urozgan in 2024, with 23.45, and 2 consecutive years in Kandahar, with 19.16 and 19.91, respectively. In terms of adequate investigation in the 48-hour time frame, the overall proportion was 98.3% for both years, with all of the provinces reporting more than 95% during 2024-2025. Similarly, the specimen adequacy proportion was 99% and 99.2% in 2024 and 2025, respectively, with all of the provinces of the region reporting a higher proportion than the target of 80%. However, at a regional level, the timeliness of specimen shipment to the laboratory and its receival during 5 days was 77.7% in both years, with a slightly lower proportion in 2025 (75.9%). In comparison, at the provincial level, only Kandahar (72.5%) and Farah (68.8%) had a lower proportion for this indicator compared with four other provinces, which all reported achieving target proportion of 80%. Moreover, reporting to the National Expanded Program on Immunization was used as a proxy for the indicator of IgM results reported to public health authorities, which illustrated an overall proportion of 82.8%, with an annualized report of 78.9% in 2024 and 86.2% in 2025. Helmand, Urozgan, and Nimroz reported a lower proportion for this indicator across both years against the 80% target.

**Table 5:** Measles surveillance system performance by the provinces of the southern region, 2024-2025.

Indicator	Target	Period	Kandahar	Helmand	Urozgan	Zabul	Farah	Nimroz	Total
Discarded case rate (per 100,000)^b^	≥2.00	2024	19.16	6.69	23.45	5.43	1.01^a^	1.55^a^	11.29
		2025	19.91	4.46	13.82	5.18	1.35^a^	1.03^a^	9.81
		**2024–2025**	**19.54**	**5.57**	**18.64**	**5.31**	**1.18**^a^	**1.29**^a^	**10.55**
Adequate investigation ≤48 hours, excluding travel history^c^	≥80%	2024	97.4	100.0	99.7	99.3	100.0	100.0	98.3
		2025	98.5	96.5	100.0	97.9	97.3	96.0	98.3
		**2024–2025**	**98.0**	**98.4**	**99.8**	**98.5**	**97.9**	**97.0**	**98.3**
Adequate specimens collected and tested^d^	≥80%	2024	99.4	98.0	99.4	97.0	90.9	100.0	99.0
		2025	99.6	97.7	98.6	96.5	100.0	100.0	99.2
		**2024–2025**	**99.6**	**97.8**	**99.1**	**96.7**	**97.9**	**100.0**	**99.1**
Specimens received at laboratory ≤5 days	≥80%	2024	73.7^a^	87.4	91.9	86.6	72.7^a^	100.0	79.8^a^
		2025	71.5^a^	89.5	84.0	89.8	67.6^a^	100.0	75.9^a^
		**2024–2025**	**72.5**^a^	**88.3**	**88.9**	**88.2**	**68.8**^a^	**100.0**	**77.7**^a^
IgM results reported ≤4 days of receipt^e^	≥80%	2024	81.1	73.8^a^	78.5^a^	72.0^a^	81.8	62.5^a^	78.9^a^
		2025	90.3	75.1^a^	69.3^a^	84.3	94.6	75.0^a^	86.2
		**2024–2025**	**86.4**	**74.4**^a^	**74.9**^a^	**78.3**^a^	**91.7**	**71.4**^a^	**82.8**

Abbreviations: IgM, immunoglobulin M; WHO, World Health Organization.

Values are percentages, except indicator 2 (discarded cases per 100,000 population; 2024-2025 annualized).

a Below the WHO target. Numerators and denominators are provided in Supplementary Table S1. Seven cases residing outside the catchment provinces (Herat, Ghor, and Kabul) are included in the total for indicators 3-7. Indicators 1, 5, and 8 were not assessed.

b Final classification “discarded”; provincial populations applied to both years (total = 4,667,281). Strict definition (also rubella IgM-negative): 10.21 per 100,000.

c Investigation 0-2 calendar days after notification, with district, age, sex, rash onset date, specimen collection date, and vaccination status recorded.

d Blood specimen collected 0-28 days after rash onset, adequate condition, with a measles IgM result.

e Date report to National Expanded Program on Immunization (NEPI) was used as a proxy for the date when the results were reported to public health authorities.

## Discussion

### Main findings

The results of our analysis among 4379 suspected cases of measles in southern region of Afghanistan during 2024-2025 highlights a measles epidemic with a higher burden among young, unvaccinated children, with an extreme burden of children not receiving MCV, while highlighting surveillance performance. The authors found that almost four-fifth of the of the cases were aged under 5 years, with almost similar sex-based distribution. An increase of 16% was observed during 2025 compared with the previous year. Our findings suggest a near-universal presence of children with no history of MCV administration in this specific cohort. Only one in 10 suspected case has a history of receiving one dose of the MCV. Geographical disparities in MCV reported history of suspected cases were also reported in our study, with a higher proportion recorded in Kandahar compared with Zabul, Urozgan, and Helmand. From the overall eight indicators of surveillance performance assessment recommended by the WHO, this study analyzed five indicators (where data allowed). The region’s case-based surveillance performance scored higher than the recommended target in four of five selected indicators, with specimen receipt at the laboratory in ≤5 days scoring slightly lower than the target.

### Comparison with the literature

Distribution of suspected cases of measles in the southern region of Afghanistan is consistent with the regional landscape. The analysis of measles cases across the EMR during 2019-2024 shows the burden of this infection among children aged under 5 years at 72%, which is comparable to our findings [9]. The 14% proportion of suspected cases among children aged under 9 months in our study is lower than previous observations in Afghanistan, which elucidated 43.5% of suspected cases in this age category. However, the comparison should be made with caution because the previous observation was based on outbreak reports compared with the current study, which focuses on case-based surveillance [12]. In terms of sex-based distribution of the cases, our findings from the southern region is similar to the NDSR national cumulative incidence report, highlighting slight predominance of males in the number of suspected cases [19]. Comparing with neighboring countries, a descriptive study in Pakistan reported measles cases in the age group of under 9 months at roughly double the proportion observed in our study, with 30% of their overall measles cases occurring in this age group compared with 14% in our study [20]. Moreover, the elevated proportion of cases aged under 9 months found in our study is consistent with recent WHO analyses of global case-based surveillance data (2012-2023), which found that age-specific measles incidence rates were typically highest among children aged 6-8 months and 9-11 months in the African, Eastern Mediterranean, and Southeast Asian regions, with these rates increasing further during 2022-2023 [21]. On the other hand, these findings are consistent with modeled Rcov from MICS 2022-2023 in the southern region, reflecting the high-risk transmission status of the mentioned provinces [15]. A major finding of this study is the high burden of suspected cases with no reported history of MCV uptake, which consisted of 80.9% of the overall cases. This vaccination gap was previously recorded in the 2019-2021 and 2021-2023 outbreaks, which showed 98.5% and 97.1%, respectively [12]. From a broader perspective, immunization coverage is not a southern region–specific obstacle but a national concern, despite its improvements. Against the WHO-recommended threshold of 95% coverage for two doses of MCV, the MICS results suggest 51% and 36.8% of coverage, respectively [14]. On the other hand, a survey of immunization coverage in 13 urban areas in Afghanistan supports this concern, reporting only 53% of MCV1 coverage [22]. On a further note, the prevalence of suspected cases with no reported history of MCV vaccination shows varying distribution province-wise, with a higher proportion reported from Urozgan, Zabul, and Helmand, provinces with an estimated higher Rcov and measles virus transmission [15]. Although a nationwide campaign reached the provinces of the southern region in 2025 during the second phase, the lack of published data on province-wise pre- and post-campaign immunization data hindered the comparison and an outlook of progress, whereas the campaign was completed in mid-November 2025 [13]. Several reasons may contribute to this high level of no-MCV status, including but not limited to sociocultural barriers and vaccine hesitancy with widespread rumors and myths regarding vaccines; issues in terms of accessibility of health services and sex disparities are also worth of considering [7,16,[23], [24], [25]]. The mentioned factors are also observed in other settings and in wider range at a global level, with multifaceted reasons fueling the no-vaccination status worldwide [26]. The findings regarding the immunization status of suspected cases and their comparison with MICS and other published works should be made with caution because administrative data of routine immunization is not accounted in this comparison.

The case-based surveillance system for measles in the southern region of Afghanistan assessed during this study’s time frame was optimal in four of five of the selected indicators. The observed discarded case rate of 10.55 per 100,000 population was substantially higher than the WHO-recommended two per 100,000 population, suggesting that the surveillance system in the southern region is appropriately discarding non-measles febrile rash illness, highlighting strong surveillance sensitivity. Results from the 10-year surveillance evaluation in Babol, Iran, neighboring Afghanistan, showed a rate of 6.26 per 100,000, which is still lower than what was observed in this study [27]. On the other hand, countries with similar epidemiologic profiles to Afghanistan such as Nigeria showed that the country never reached the WHO-recommended target of two per 100,000 population across an 8-year surveillance. Moreover, the surveillance’s performance in timely case investigation (at 98.3%) in our study was comparable to the findings from Iran [27] and the 5 year surveillance data of Ghana [28]. The illustrated proportion of 99.1% suspected cases with adequate specimen collected and tested was well above the WHO-recommended target of 80%, contrasting with the 41.5% of East Java, Indonesia [29], considering the limitation in reagent stocks. On a broader level, this Afghanistan-level result is above the WHO African Region analysis of a 4-year surveillance, which highlighted that only 56% of suspected cases had specimens collected for diagnostic processing [30]. Although specimen receipt of 77.7% in our study fell short of the WHO-recommended target, the difference was not showing substantial gaps. In comparison, the proportion of specimens arriving at the laboratory over 8 years in Nigeria was only 54%, considering that the indicator was set for 3 days in that setting [31]. In addition, the proportion of IgM results communicated with public health authorities found in our study meets the target with only 82% but falls short of the findings from Iran that showed 95-98% [27]. The selected settings as comparators are based on the availability of published literature on performance of case-based measles surveillance assessment and/or measles surveillance evaluation while sharing some epidemiologic and socio-economic similarities. Moreover, Afghanistan shares the WHO EMR with Iran.

## Conclusion

This 2-year surveillance analysis of measles in the southern region of Afghanistan demonstrated a heavy burden concentrated among young, unvaccinated children, with a near-universal burden of cases with no recorded history of MCV (both doses) across all six provinces and pronounced provincial disparities in vaccination status and surveillance timeliness. These findings reinforce that the region’s measles burden follows the broader national pattern and is plausibly linked to the sociocultural and programmatic barriers to routine immunization documented elsewhere in Afghanistan. The province-specific and surveillance-strengthening priorities outlined previously warrant urgent, coordinated action to curb the region’s measles resurgence.

### Policy implications

The overwhelming burden of suspected cases with no record of MCV in some southern region provinces calls for province-specific strategies and tailored intervention to increase the demand and improve the routine immunization coverage. Implementing SIAs, reflecting importance of immunization through social leaders, village heads (Maliks), and religious scholars would all help to improve the routine immunization coverage. On the other hand, the presence of measles cases in a significant proportion of children aged under 2 years children calls for a holistic approach that goes beyond immunization and address underlying issues in nutrition and maternal health as well. Moreover, addressing sociocultural barriers, such as misconceptions about vaccination, may potentially lead to a decrease in vaccine hesitancy and eventual reduction in the unvaccinated children burden. Community engagement programs at the provincial and district level, with integration of community members, religious scholars, and other influential individuals in the provincial level, could be helpful in tackling the vaccine hesitancy. On the theme of surveillance system performance, disproportionate timeliness performance among provinces requires interventions, such as enhancing cold chain courier networks between regional laboratories and provinces to improve the low-performing provinces. The overall discarded rate across 2 years of the study period reflects a positive trend in surveillance system sensitivity, which could be sustained through continuing filed supervision and case investigation capacity building.

### Limitations

The authors acknowledge four limitations in this study. First, the case-based surveillance for measles, which was used in this study, is contingent on the completeness and quality of routine reporting. Under-reporting may have influenced the actual measles burden, which could be higher than what has been reported. Second, the immunization status of cases was retrieved through card verification or recall, which introduced recall bias. Hence, the vaccination history data should be interpreted cautiously in this study. Third, the denominators for the surveillance system performance indicators were used based on the NSIA estimates that may not reflect the accurate number. Finally, outcome data were not collected adequately across both years and hence was not entered into the analysis, which limits the ability to draw conclusion about case-fatality rate, complication burdens, or clinical severity at the population level.

## Funding

This research did not receive any specific grant from funding agencies in the public, commercial, or not-for-profit sectors.

## CRediT authorship contribution statement

**Shoaib Naeemi:** Writing – review & editing, Writing – original draft, Supervision, Software, Methodology, Formal analysis, Data curation, Conceptualization. **Mustafa Rahimi:** Writing – original draft, Project administration, Data curation, Conceptualization. **Nisar Ahmad Niazi:** Writing – original draft, Project administration, Data curation, Conceptualization. **Rohullah Sakhi:** Writing – review & editing, Writing – original draft, Software, Formal analysis.

## Declaration of competing interest

The authors have no competing interests to declare.

## Appendix Supplementary materials

Image, application 1
