# Supplementary material for: Descriptive epidemiology of measles in the southern region of Afghanistan, 2024-2025: a secondary analysis from regional case-based surveillance data
Source: IJID Reg. 2026 Oct 4;21:101036. doi: 10.1016/j.ijregi.2026.101036 (PMC13743472; doi:10.1016/j.ijregi.2026.101036)
Supplement: Supplementary file 1 [file mmc1.docx]

**Supplementary Material**

**Supplementary Figure 1**

*Descriptive Epidemiology of Measles in the Southern Region of Afghanistan, 2024–2025: A Secondary Analysis from Regional Case-Based Surveillance Data*

|  | **STEP 1**  **Patient with rash illness presents to health facility** |  |
| --- | --- | --- |
|  | **↓** |  |
|  | **STEP 2**  **Clinical assessment by attending medical doctor at health facility** |  |
|  | **↓** |  |
|  | **DECISION**  **Does the case meet the national measles case definition?**  **(Fever + maculopapular rash + cough and/or coryza and/or conjunctivitis)** | **No 🡪 Case does not meet case definition**  **Manage other illnesses**  **Report as non-measles rash illness** |
|  | **↓ YES** |  |
|  | **STEP 3**  **Case entered into measles line-list**  **Alert classification assigned**  **Contact tracing initiated** |  |
|  | **↓** |  |
|  | **STEP 4**  **Venous blood specimen collected at health facility** |  |
|  | **↓** |  |
|  | **STEP 5**  **Specimen transported to**  **Kandahar Regional Reference Laboratory (KRRL)** |  |
|  | **↓** |  |
|  | **STEP 6 \| LABORATORY TESTING**  **Measles IgM ELISA performed on all specimens**  **Rubella IgM tested if measles IgM negative / equivocal or rubella suspected** |  |
|  | **↓** |  |
| **STEP 7 \| LINE-LIST UPDATED**  **Laboratory results received → Line-list updated with final case classification** | | |
| **IgM Positive** | **IgM Negative** | **Rubella IgM Positive** |
| **LABORATORY-CONFIRMED MEASLES** | **DISCARDED**  **(Not measles / Not rubella)** | **LABORATORY-CONFIRMED RUBELLA** |
| **↓** |  |  |
| **STEP 8 \| CASE FOLLOW-UP**  **Surveillance team follows up to ascertain final outcome of confirmed cases** |  |  |
| **↓** |  |  |
| **FINAL OUTCOMES (reported in line-list)** | | |
| **RECOVERY** | **DEATH** | **REFERRAL WITH COMPLICATION (to higher-level health facility)** |

*Supplementary Figure 1. Measles Case-Based Surveillance Pathway, Southern Region of Afghanistan. KRRL = Kandahar Regional Reference Laboratory; IgM = Immunoglobulin M; ELISA = Enzyme-Linked Immunosorbent Assay.*

| Supplementary Table 1. Monthly Distribution of Suspected Measles Cases by Year, Southern Region of Afghanistan, 2024–2025 | | | | | | |
| --- | --- | --- | --- | --- | --- | --- |
| Month | 2024 | % of 2024 | 2025 | % of 2025 | Total | % of Total |
| January | 82 | 4 | 294 | 12.5 | 376 | 8.6 |
| February | 105 | 5.2 | 328 | 14 | 433 | 9.9 |
| March | 147 | 7.2 | 341 | 14.5 | 488 | 11.1 |
| April | 145 | 7.1 | 335 | 14.3 | 480 | 11 |
| May | 196 | 9.7 | 279 | 11.9 | 475 | 10.8 |
| June | 230 | 11.3 | 29 | 1.2 | 259 | 5.9 |
| July | 258 | 12.7 | 141 | 6 | 399 | 9.1 |
| August | 195 | 9.6 | 107 | 4.6 | 302 | 6.9 |
| September | 174 | 8.6 | 118 | 5 | 292 | 6.7 |
| October | 152 | 7.5 | 99 | 4.2 | 251 | 5.7 |
| November | 158 | 7.8 | 152 | 6.5 | 310 | 7.1 |
| December | 187 | 9.2 | 127 | 5.4 | 314 | 7.2 |
| Total | 2,029 | 100 | 2,350 | 100 | 4,379 | 100 |
